# Representational Momentum in the Expertise Context: Support for the Theory of Event Coding as an Explanation for Action Anticipation

**DOI:** 10.3389/fpsyg.2019.01838

**Published:** 2019-08-14

**Authors:** Dior N. Anderson, Victoria M. Gottwald, Gavin P. Lawrence

**Affiliations:** School of Sport, Health and Exercise Sciences, Institute for the Psychology of Elite Performance, Bangor University, Bangor, United Kingdom

**Keywords:** representational momentum, expertise, action anticipation, theory of event coding, rugby

## Abstract

This study aimed to extend the notion of the theory of event coding as an explanation of action anticipation in expert sport performers. This was achieved by investigating the degree with which automatic anticipation depends on the ecological congruency between the perceived action and its distal effect. In a novel approach, the representational momentum paradigm was adopted to address this notion. Expert (*N* = 16) and novice (*N* = 20) rugby players observed a dynamic video of a short pass that was displayed as either toward or away from a receiver. Following an occlusion interval, participants were required to judge whether the video resumed at the same place, further forward or further backward than its original stopping place. Experts demonstrated stronger anticipatory tendencies when the action was directed toward the receiver. This relationship was modulated by a leftward directional bias that is discussed in the context of a bias in viewing behavior that is underpinned by attention. Novice anticipatory tendencies were independent of context. These findings show support for the extension of the theory of event coding.

## Introduction

Conventional accounts of motor control theory have linked the afferent-efferent coding of motor actions to that of their respective outcomes (Adams, 1971; Schmidt, 1975; Prinz, 1997; Hommel et al., 2001). Indeed, strong associations between the quality of motor actions and their respective effects characterize sporting expertise. Research has demonstrated that, compared to novices, expert sport performers demonstrate superior ability in the planning and execution of actions in relation to their distal effects (e.g., Ford et al., 2006). Accordingly, experts find themselves exposed to competitive scenarios in which the successful anticipation of another performer’s action is paramount for successful performance (Williams, 2009). In the current study, the researchers investigated the extent that the superior ability demonstrated by experts is dependent on the ecological congruency between the observed action and its anticipated effect.

Hommel et al. (2001) proposed the theory of event coding (TEC). The TEC offers a framework for the cognitive underpinnings of action planning and perception and could serve as a sound explanation for anticipatory behavior of expert performers. The TEC proposes that overlapping features in sensorimotor representations reflect the common coding of both actions and their perceived distal effect; when it comes to action planning, it means that once such action-effect binding has occurred, one only has to anticipate the effect to trigger the execution of the associated action (Hommel et al., 2001). Accordingly, supporting evidence for the TEC also suggests that experience in action planning facilitates the tendency to anticipate similar actions of others toward distal effects when these actions are congruent with those previously learned (Knoblich and Flach, 2001; Wilson and Knoblich, 2005; Prinz, 2006). This suggests that experts acquired a form of anticipatory behavior that is developed as a function of action planning experience, a notion which has been frequently observed in paradigms that have reported expert-novice differences in anticipatory behavior across a variety of sporting expertise domains (Cañal-Bruland et al., 2011; Vicario et al., 2017; Murphy et al., 2018).

In light of this, one can assume that for anticipation of an action to occur, features in the observer’s visual field must be congruent with the ecological features of the action, such that a likely distal effect in the environment can be commonly coded with the action as it is observed. These features could take the form of appropriate physical space in the direction of action, or the presence of a landmark, such as a receiving actor or a contextually relevant target. Conversely, under conditions in which the features that the observer perceives are incongruent with the ecological features of the action, (according to the premise of the TEC) anticipation of the action would likely be compromised. This study aimed to test this proposition by comparing the anticipatory behavior of experts in response to observing an action that is performed with an ecologically congruent landmark in the observer’s visual field versus an action that is ecologically incongruent with the visual field landmark. To test for the effect of expertise, we also observed the anticipatory behavior of novices in response to the same set of stimuli.

A measure of automatic anticipatory behavior that is widely used in the perception literature stems from the representational momentum (RM) phenomenon (see Hubbard, 2014 for a review). RM describes the local, bottom-up nature of motion perception and is defined as the tendency to extrapolate the position of an object beyond that last observed. In Freyd and Finke’s (1984) original paradigm, participants were required to view a sequence of three images of a rectangle, each image indicating an implicit direction of the rectangle’s rotation. Participants were instructed to remember the orientation of the third rectangle prior to the presentation of a fourth that was either in the same position as the third, rotated in the implicit direction, or rotated in the reverse direction. When the inducing sequence implied a consistent direction of motion, participants declared the position of the fourth rectangle as being in the same position as the third rectangle when it had actually moved forward. This finding suggested that participants extrapolated the position of the third rectangle beyond that induced by the display sequence, demonstrating the RM effect.

The RM effect has received widespread empirical support across a range of experimental stimuli, such as apparent motion in static images (Senior et al., 2000), smooth continuous vertical and horizontal motion (Hubbard and Bharucha, 1988; Gottwald et al., 2015), biological motion (Verfaillie and Daems, 2002; Wilson et al., 2010), and motion-induced real-world scenes from the first-person view (Thornton and Hayes, 2004; Blättler et al., 2010, 2011). Of particular interest to the current study is the modulating role that sporting expertise plays in the underlying memory distortions associated with the RM effect. Specifically, expectations derived from an observer’s domain of sporting expertise have been shown to influence the RM effect (Didierjean and Marmèche, 2005; Gorman et al., 2011). For example, Gorman et al. (2011) investigated the influence of sporting expertise on complex pattern recall using the RM paradigm. Expert and novice basketball players observed an inducing display of a complex basketball scene and were asked to recall the position of its interrelated elements after a brief interval. Findings showed that although both groups demonstrated the RM effect when the display contained motion, only the experts demonstrated increased anticipatory tendencies for the static images. The authors attributed these findings to a deeper understanding of the associations between the elements in the display within the expert players.

In light of previous findings, the current aim was to further explore the proposed common coding of actions and their distal effects (as outlined by the TEC) in the context of automatic action anticipation. Specifically, this study observed the extent with which expertise in rugby elicited the RM effect when observing a domain-specific motor action that was either ecologically congruent (i.e., directed toward a typically expected outcome) or ecologically incongruent (i.e., directed away from a typically expected outcome). Novice and expert rugby players observed the initiation of the short pass, orientated either toward (congruent) or away (incongruent) from a receiver. After a short occlusion interval, the display then re-appeared at one of several test frame positions that either induced reversed, forward, or no momentum displacement. Participants were required to make judgments as to whether they perceived the test frame position as being shifted backward, forward, or in the same position as the last frame prior to the occlusion interval.

We hypothesized that expert participants would have developed a strong encoding of action-effect relationships, and therefore expected them to produce stronger memory distortions when the observed action was directed toward a receiving player. When the short pass was directed away from the receiver, however, it was expected that experts would not demonstrate anticipatory tendencies, and thus memory distortions in this condition would not occur. In contrast, we did not expect novices to have acquired the same action-effect encoding, and thus we did not expect any anticipatory tendencies observed to be influenced by ecological validity, and therefore expected memory distortions not to interact with the congruency of actions directed either toward or away from the receiver.

## Materials and Methods

### Participants

Expert (*N* = 16; mean age 22.61 ± 1.67 years) and novice rugby players (*N* = 20; mean age 21.42 ± 2.12 years) provided informed consent to participate in the study. All were naïve to the research hypotheses being tested. Procedures were carried out in accordance with the ethical guidelines outlined by the Ethics Committee of the School of Sport, Health and Exercise Sciences, Bangor University for research involving human participants and in line with the 1964 Helsinki declaration and its later amendments or comparable ethical standards. Participants were classified into their respective expertise groups according to number of years playing rugby and time currently spent training and competing on a weekly basis. As such, the expert group had accumulated an average of 7.30 ± 3.52 years of rugby-specific playing experience at amateur club level, and were all currently training and competing for 5.46 ± 4.51 h per week. Conversely, the novice group had accumulated an average of 0.16 ± 0.53 years playing experience, and none were currently training or competing in rugby.

### Apparatus

**Figure 1:**
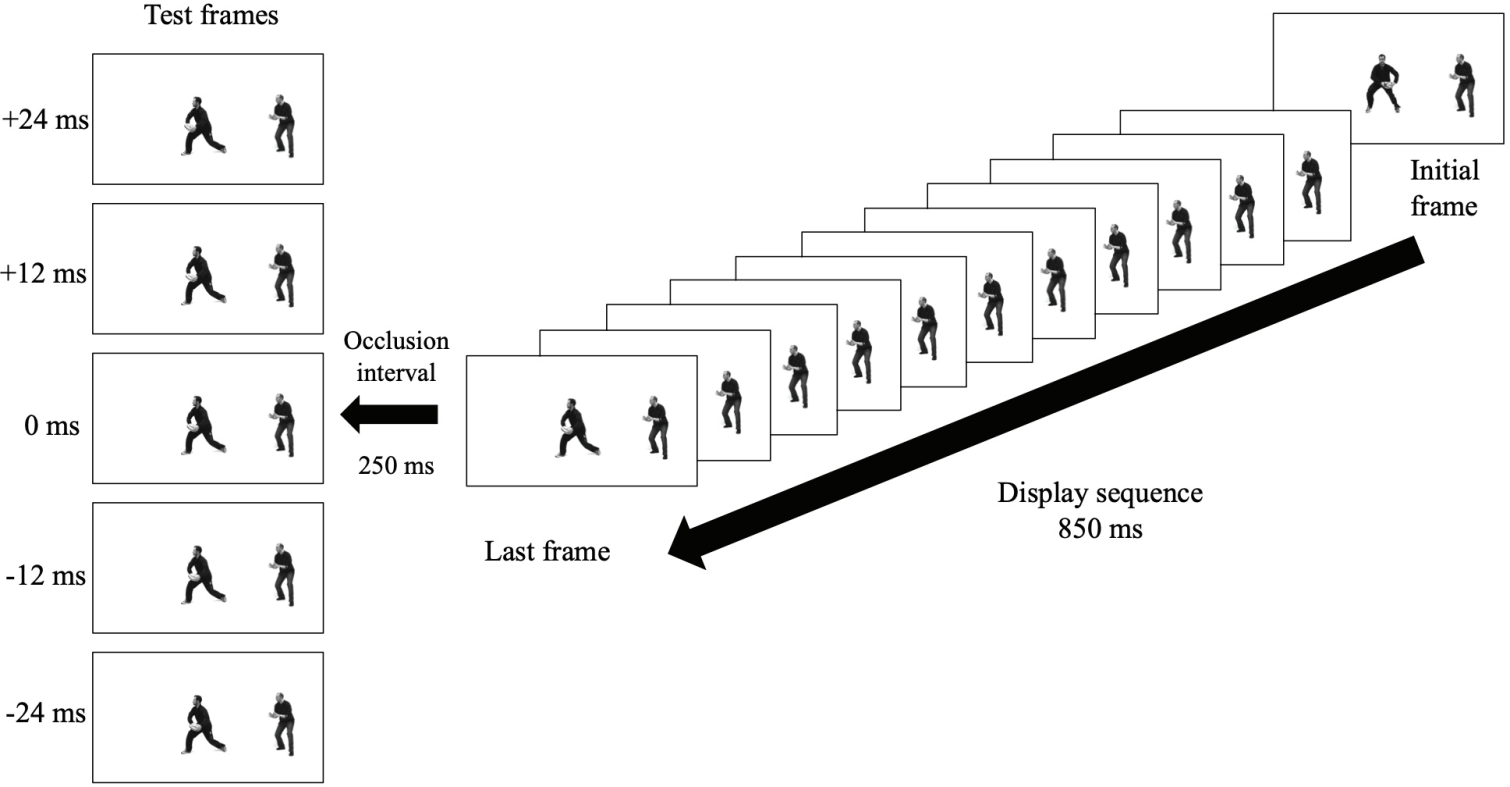
A schematic representation of a trial in the leftward “away” condition. Both the passer and receiver appeared at the same time in the initial frame. The sequence then displays the series of images depicting the passer performing the action either toward or away from the receiver for 850 ms to the position of the last frame prior to disappearing for an occlusion interval of 250 ms and then re-appearing at one of five test frames.

The video was recorded using a Basler™ high-speed camera (160 frames per second). During recording, the passer was instructed to execute a simple pass to his right toward the receiver, with the pass executed over a distance of 2 m. The video was then broken down forming 100 separate frame-by-frame images of both the passer and the receiver. Each frame was edited using DirectDraw 7™ software in which passer and receiver images were mirrored to simulate the apparent motion of the action being performed in either rightward or leftward directions. Each frame was then pieced back together to simulate the display video sequence (see Figure 1) using Visual Basic 6™ software running at a refresh rate of 85 Hz. Four separate sequences were created to simulate the passer initiating the pass either toward or away from the receiver in both directions. The display was presented on a DELL™ Trinitron UltraScan P991 monitor (85 Hz) with a 1,600 × 1,200 resolution running from a desktop computer (Compusys Computers Ltd. Core 2 Duo 3 GHz, 2 GB RAM, ATI 128 MB Graphics card, Windows XP operating system). Participants sat at a distance of 1 m from the monitor with their head fixed on a chin rest in order to ensure the participants maintained their viewing position and visual angle. A response keypad with three active keys (1 = same, 2 = forward, 3 = reverse) was connected to the computer in order for the experimenter to register the participants’ verbal response for each trial.

### Task and Procedure

For each trial, participants were presented with the video representing the initiation of the short pass orientated either toward (congruent) or away (incongruent) from a receiver (see Figure 1). This video lasted approximately 850 ms prior to disappearing for an occlusion interval of 250 ms. This occlusion interval is in line with previous RM paradigms (e.g., Wilson et al., 2010) and was chosen to preserve the RM effect, which has been shown to decay after a few hundred milliseconds (Freyd and Johnson, 1987; Bertamini, 1993). To prevent participants from memorizing the position of the final frame in the sequence, half of the trials disappeared at the 99th frame in the video, and the other half at the 100th frame. The display then re-appeared at one of the five test frame positions indicating different temporal shifts in the display sequence corresponding to the displacement momentum and the number of frames displaced (i.e., −24, −12, 0, +12, +24 ms; with each frame shift representing a 12-ms temporal shift). On presentation of the test frame, participants were asked to verbally respond to whether they perceived the test frame position as shifted forward, backward, or in the same position as the last frame prior to the occlusion interval. The experimenter, who was blind to the presentation of the stimulus, then documented the participant’s verbal response onto the computer *via* the response keypad. Participants were asked to respond as quickly and as accurately as possible in order to ensure the responses were based on the first impression of the stimulus. This was especially important as the RM effect is a measure of automatic anticipation, which has been shown to decay with longer response times. The testing phase consisted of 240 trials {six random repetitions of each of the possible 40 trial combinations [2 conditions (toward, away) × 2 directions (leftward, rightward) × 2 last frame positions (99th, 100th) × 5 test frame positions]}.

### Dependent Measures and Analyses

#### Weighted Means

**Figure 2:**
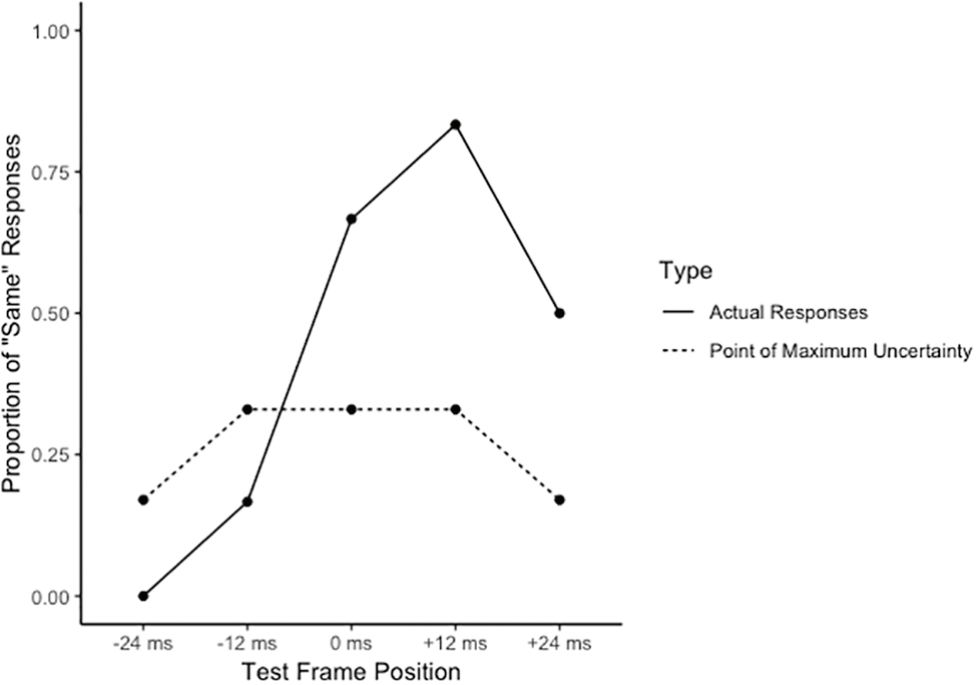
A case example of the distribution of the proportion of “same” responses across each test frame position. The dashed lines represent the point of maximum uncertainty for the “same” responses at each test frame position (note the respective decrease in the point of maximum uncertainty at the two extreme test positions. This would indicate a reduced threshold for uncertainty, or an increased expectation that participants will be more certain about their response in these test frame positions).

To attain a measure of the RM effect, we calculated an adapted version of the weighted means as used in many RM paradigms (e.g., Munger, 2015). Specifically, the proportion of “same” responses at each test frame position was subtracted from the relative point of maximum uncertainty and was then summed across all test frame positions. The relative point of maximum uncertainty was determined as the rate at which a given response would indicate uncertainty about the position of a test frame. This was expected to be equally distributed for each response for the 0-ms test frame (i.e., 1/3 responses = 0.33 for “forward,” “same,” and “reverse” responses), and disproportionately distributed in all other test frame positions as a function of the product of the number of potential responses [i.e., 3 (“forward,” “same,” or “reverse”)] and the number of frames the test frame had shifted from the final position of the video. For example, the point of maximum uncertainty for “same” responses in the +24-ms test frame (i.e., +2 frames) would be 1/(3 responses × 2 frame shifts) = 0.17. See Figure 2 for a breakdown of a case example.

The proportion of response rates at each test frame was subtracted from its relative point of maximum uncertainty, multiplied by a position weight of −1, 0, or 1 (for respective reversed, same, or forward test frames), and then summed to produce a weighted value. A positive value would indicate that the test frames that had actually shifted forward (i.e., the +12-ms and +24-ms test frames) were incorrectly identified as “same,” therefore indicating the presence of the RM effect. In order to appropriately weigh this value for each condition, we divided this summed value for each participant in each condition by the mean of the overall summed value for all participants across all conditions. These values were then subjected to a 2 (group: experts, novices) × 2 (condition: toward, away) × 2 (direction: left, right) mixed model ANOVA.

#### Response Error

To assess the overall accuracy of the responses, and thus the perceptual sensitivity within participants, we recorded the percentage of incorrect responses at each test position and subjected mean values to a 2 (group) × 2 (condition) × 2 (direction) × 5 (test frame) position ANOVA with repeated measures on the last factor. Sphericity corrections were applied to the model using the Greenhouse Geiser adjustment.

## Results

### Weighted Means

**Figure 3:**
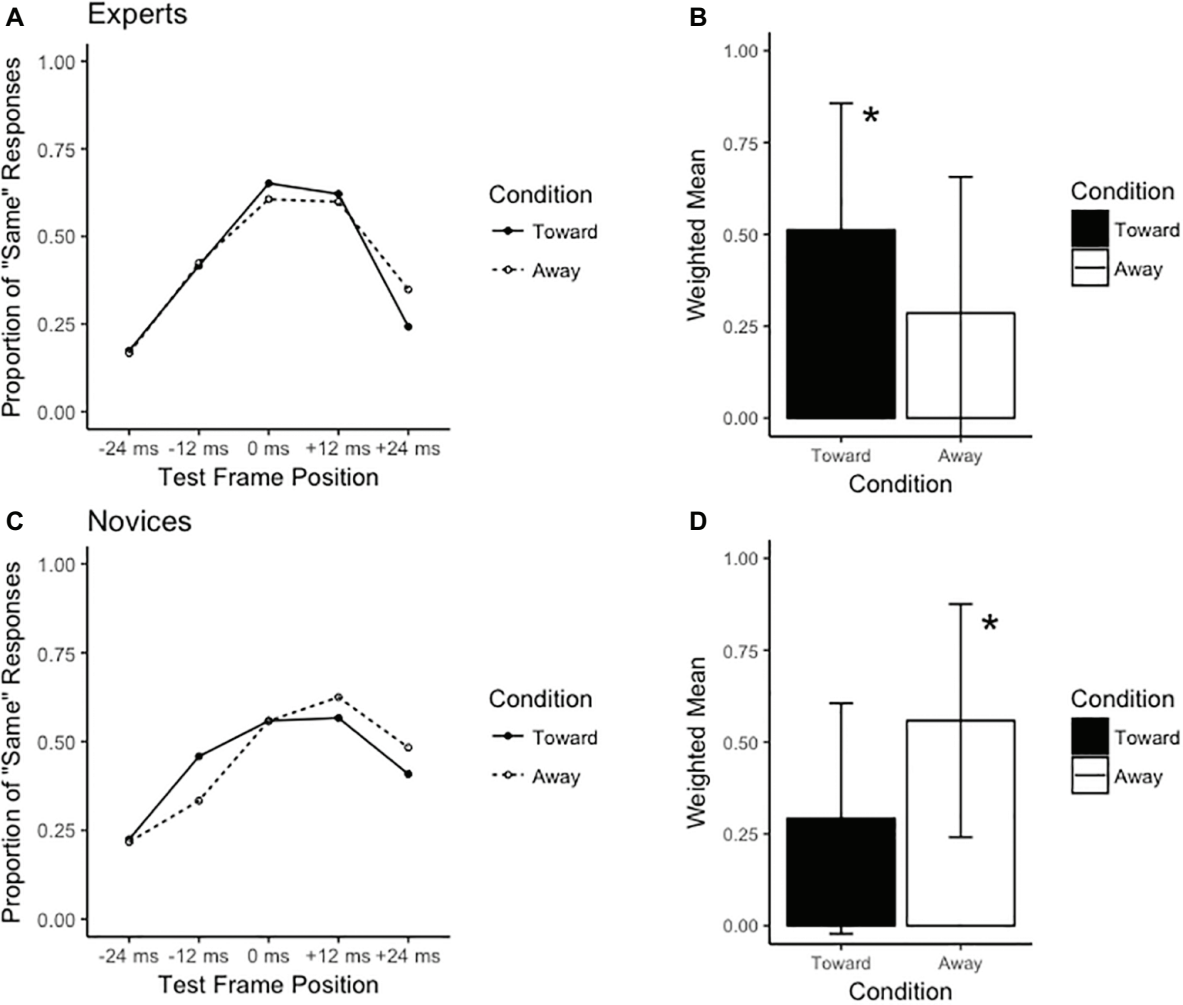
Results for the leftward direction. The left-hand side of the figure displays the distribution of the proportion of “same” responses across test frame positions for **(A)** experts and **(C)** novices. The right-hand side displays the weighted means for **(B)** experts and **(D)** novices. * indicates significant difference from “away” condition at *p* < 0.05.

**Figure 4:**
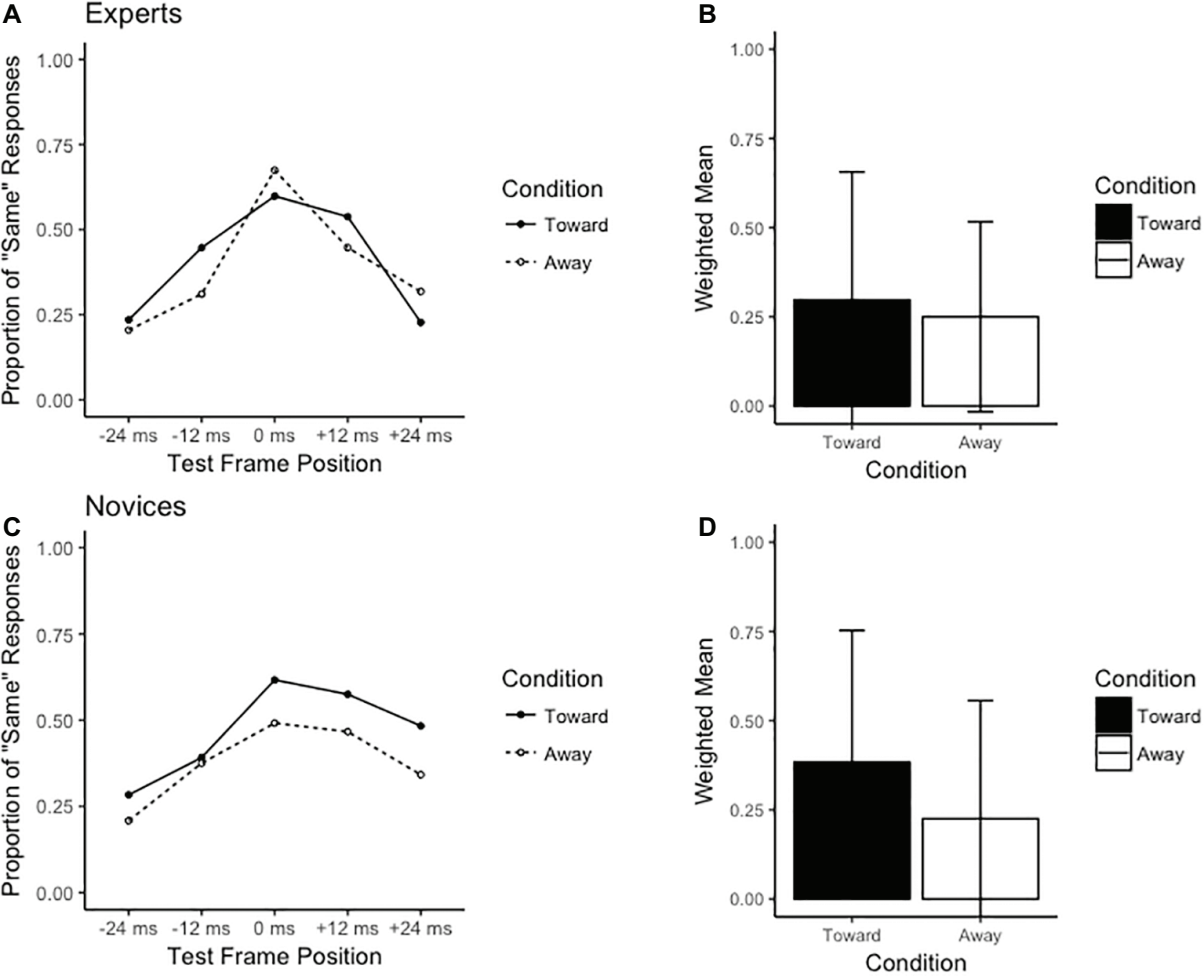
Results for the rightward direction. The left-hand side of the figure displays the distribution of the proportion of “same” responses across test frame positions for **(A)** experts and **(C)** novices. The right-hand side displays the weighted means for **(B)** experts and **(D)** novices.

The ANOVA revealed non-significant main effects for group (*F*_1,35_ = 0.11, *p* = 0.74, *ηG*^2^ = 0.00), condition (*F*_1,35_ = 0.11, *p* = 0.75, *ηG*^2^ = 0.00), and direction (*F*_1,35_ = 2.25, *p* = 0.14, *ηG*^2^ = 0.01). However, a significant group × condition × direction interaction was observed (*F*_1,35_ = 8.99, *p* < 0.01, *ηG*^2^ = 0.01). Breakdown of this interaction *via* separate 2 (group) × 2 (condition) ANOVAs for each direction revealed a group × condition interaction when the stimulus was directed to the observer’s left (*F*_1,35_ = 8.63, *p* < 0.01, *ηG*^2^ = 0.03; see Figure 3), but not the observer’s right (*F*_1,35_ = 1.60, *p* = 0.21, *ηG*^2^ = 0.00; see Figure 4). *Post hoc* inspection using Tukey’s HSD revealed that while novices observed an RM effect in both the congruent (toward) and incongruent (away) conditions, experts produced significantly greater RM effects (higher weighted mean values) in the congruent compared to incongruent conditions (*t*_15_ = 1.75, *p* < 0.05, *d* = 0.36) in the leftward direction. Additionally, the weighted mean value significantly differed from 0 for experts in the congruent condition only (*t*_15_ = 2.69, *p* < 0.01, *d* = 0.86; see Table 1).

**Table 1:** Weighted means (and standard deviations) for each group in each experimental condition. Bold font values indicate expert values that are significantly different from respective novice values.

	Leftward				Rightward			
	Toward		Away		Toward		Away	
Novices	0.83	(1.90)	1.58	(1.92)	1.09	(2.24)	0.64	(2.01)
Experts	**1.45**	(1.69)	**0.81**	(1.82)	0.84	(1.76)	0.71	(1.31)

The data here present a rather unexpected finding: the expertise-induced RM effect seemingly operated as a function of a directional bias, specifically a leftward bias. Expert rugby players were able to extrapolate, and thus anticipate, the movement of the passing player beyond its last observed position when the pass was congruent (i.e., directed toward the receiver), but only when this action occurred to the left. When the pass was incongruent by being directed away from the receiver or was directed rightward, experts were able to more accurately recall the movement of the passing player, and thus did not demonstrate any anticipatory behavior. Novices did not appear to differentiate the congruency of context since they demonstrated memory extrapolations independent of the pass being directed toward or away from the receiver.

### Response Error

Data for leftward and rightward direction stimuli are presented in Tables 2 and 3, respectively. As expected, the analysis revealed a main effect for test frame position (*F*_4,128_ = 21.718, *p* < 0.001, *ηG*^2^ = 0.22), with the error rate at test position −24 ms being significantly lower than all other test positions (*M* = 0.27, *S* = 0.29; relative to test frame 0 ms: *t*_135_ = −4.113, *p* < 0.001, *d* = 0.47), and the error rate at test position +12 ms being significantly higher than all other test positions (*M* = 0.69, *S* = 0.25; relative to test frame 0 ms: *t*_135_ = 9.759, *p* < 0.001, *d =* 1.14). A test frame position × condition interaction was also observed (*F*_4, 128_ = 3.441, *p* < 0.05, *ηG*^2^ = 0.007), with error rates in the congruent condition (*M* = 0.75, *S* = 0.23) being higher than the incongruent condition (*M* = 0.63, *S* = 0.25) at test frame position +12 ms (*t*_67_ = 4.311, *p* < 0.001, *d =* 0.5). Finally, a significant group × direction × condition × test frame position interaction was observed (*F*_4,128_ = 3.548, *p* < 0.05, *ηG*^2^ = 0.009). Breakdown revealed that error rates in the incongruent condition at test frame position 0 ms were significantly lower for experts (*M* = 0.26, *S* = 0.25) compared to novices (*M* = 0.51, *S* = 0.21) (*t*_66_ = −2.44, *p* < 0.01, *d =* 1.09; see Table 3). In the congruent condition, error rates were lower at test frame +24 ms for experts (*M* = 0.29, *S* = 0.25) compared to novices (*M* = 0.60, *S* = 0.31) (*t*_66_ = −3.277, *p* < 0.01, *d* = 1.03). This finding is an interesting one, and suggests that experts were more sensitive to the positioning of the test frame relative to its last observed position than novices, and that this sensitivity seemingly operated at different test frame positions, depending on the orientation and direction of the observed action. It appeared that, when the pass was directed rightward and away from the receiver, experts demonstrated the tendency to more accurately recall the test frame position. However, when the pass was directed rightward and toward the receiver, experts demonstrated an increased sensitivity to the test frame position compared to novices.

**Table 2:** Response error rates (and standard deviations) for each group in each test frame position in the leftward direction.

	Leftward				
	Test frame position				
	−24 ms	−12 ms	0 ms	+12 ms	+24 ms
**Toward**
Novices	30.0 (34.0)	51.7 (0.28)	43.3 (28.3)	72.5 (23.7)	47.5 (24.9)
Experts	20.2 (28.6)	36.9 (32.1)	35.7 (28.3)	83.3 (17.2)	34.5 (28.0)
**Away**
Novices	27.5 (27.2)	42.5 (34.4)	42.5 (23.2)	70.8 (19.4)	55.0 (24.8)
Experts	19.0 (24.3)	42.8 (23.3)	38.1 (28.1)	73.8 (27.5)	32.1 (31.0)

*Rates are expressed as a percentage*.

**Table 3:** Response error rates (and standard deviations) for each group in each test frame position in the rightward direction.

	Rightward				
	Test frame position				
	−24 ms	−12 ms	0 ms	+12 ms	+24 ms
**Toward**
Novices	31.7 (33.2)	43.3 (21.9)	38.3 (26.5)	75.0 (25.0)	60.0 (30.8)
Experts	27.3 (27.4)	42.9 (26.7)	34.5 (17.9)	72.6 (28.2)	**28.6 (24.8)**
**Away**
Novices	28.3 (32.9)	46.7 (22.0)	50.8 (20.6)	56.7 (27.7)	42.5 (32.2)
Experts	23.9 (24.2)	33.3 (21.7)	**26.2 (25.1)**	53.6 (19.8)	30.9 (31.3)

*Rates are expressed as a percentage. Bold font indicates Expert values that are significantly different from respective Novice values*.

## Discussion

The current study aimed to investigate the extent with which the anticipation of an observed action is dependent on the ecological congruency between the observed action and its anticipated effect. It was proposed that due to the accumulation of domain-specific practice, expert performers would show increased automatic anticipation when observing a domain-specific action that was directed toward an ecologically congruent outcome (in the current experiment, a pass that was directed toward a team mate). Conversely, when the movement was directed away from its ecologically congruent outcome (a pass directed away from a teammate), automatic anticipation would not occur. Novices, (individuals who have not acquired these action-effect relationships) were hypothesized to have anticipatory responses independent of the ecological congruency of outcomes. The findings support our hypothesis since experts demonstrated anticipatory tendencies when observing a sport-specific action that was directed toward an ecologically congruent outcome. Moreover, when the action was directed away from its ecologically valid outcome, experts no longer extrapolated the action toward its distal effect, and thus anticipatory tendencies did not occur. Conversely, the novices did not appear to be influenced by these action-effect relationships, since their anticipatory behavior occurred independent of congruency, i.e., whether the pass was directed toward (congruent) or away (incongruent) from the receiver.

The notion of common coding of action and effects (or the TEC) has also been demonstrated empirically by Jordan and Hunsinger (2008). In a series of experiments, participants were provided with the opportunity to practice a visual tracking task before observing the same task being performed by another participant. They then compared their anticipatory tendency, measured *via* the RM effect, to a group that observed the task without acquiring prior action experience. Participants who had practiced the task produced greater RM effects than the observational group, suggesting that the learned anticipatory extrapolation occurred as a result of experiencing the action planning process, and thus its associated action-effect relationship. It must be noted, however, that the task was limited to observing the continuous motion of a stimulus on a computer monitor, without additionally processing the physical movements of the actor in control of the stimulus. In addition, only half an hour of practice was given to participants during the experimental phases, making it difficult to interpret in the context of sport performance, in which practice is usually acquired over years of experience and in which complex actions of other performers are observed. The findings of the current study support those of Jordan and Hunsinger (2008) and provide additional evidence for the TEC extending to the observation of biological motion from expert performers who have acquired action experience in field-based environments.

The findings reported in the current study are also in accordance with studies that have investigated the effect of expertise in anticipatory behavior when observing a whole-body motor task (Jackson et al., 2006; Aglioti et al., 2008; Sebanz and Shiffrar, 2009). For example, Aglioti et al. (2008) investigated expert novice differences in basketball players when observing a free throw shot. They found that experts demonstrated superior performance to novices in predicting the outcome of the shot. They also found increased motor-evoked potentials in the forearm and hand of experts when observing the basketball video clips, thereby supporting the notion of an overlap in action planning and action observation as described by the TEC. The findings of the current study support this notion of anticipatory behavior in experts by showing that the ecological congruency in the visual field of the expert must also be present in order for anticipatory behavior to occur. However, unexpectedly this effect was dependent on direction of the stimulus/video of the rugby passing action. That is, experts showed anticipatory tendencies only when the pass was congruent (directed toward the receiver) and made in the right to left direction. While this finding may be a spurious result, past research has identified similar leftward bias direction effects within RM (Hubbard and Ruppel, 1999; Ossandon et al., 2014) with effects attributed to a bias in the visuo-spatial hemisphere (Gottwald et al., 2015) and the landmark attraction effect (Hubbard and Ruppel, 1999). This landmark attraction effect discerns the salience of a physical landmark in the visual field when processing the motion of a stimulus (Bryant and Subbiah, 1994). In the present study, it is possible given the proposed bias in the visuo-spatial hemisphere (Gottwald et al., 2015) that the receiving player (i.e., the landmark) was more “noticeable” in the left compared to right visual hemisphere resulting in an increase in the anticipation of the pass when the receiver was positioned in the left visual field (as explained by TEC). This landmark attraction effect did not appear to be present in novices, who were seemingly uninfluenced by the position of the receiving player.

Results for error rates seem to suggest that experts made less error than novices when identifying the test frame position. However, this effect of expertise on sensitivity to stimuli occurred in combination with the condition and the test frame position. That is, experts demonstrated the tendency to respond more accurately to the probes that were no different to the inducing stimuli (i.e., with “same” responses), but only when the probe was directed away from the receiver. In contrast, experts were more accurate at recalling the test probe position when it had moved further along from the last frame of the video (by two frames, or approximately 24 ms and by responding with “forward”), but only when the pass was oriented toward the receiver. Taken together, these findings suggest that experts demonstrate an increased sensitivity to domain-specific stimuli compared to novices, and although they demonstrated anticipatory tendencies, these tendencies only occurred in the congruent condition. In the incongruent condition, experts showed no evidence of an anticipatory response; instead their responses met the demands of the task, which in the current study were independent of their domain of expertise, i.e., recalling the position of an inducing display. This would suggest that, when presented with stimuli in real time, the goal-directed systems would seemingly override any automatic perceptual systems, and this overriding would better operate as a function of domain-specific practice.

Although the current study reveals some interesting findings, there are some limitations. For simplicity, we restricted the stimuli presented in the study to a single action in the expertise domain. This stimuli was chosen as it represented an action that is performed on the horizontal plane and therefore limits any potential confounding effects of perceived external forces (such as gravity) on the observed RM effects. This makes it difficult to generalize current findings to actions within the same domain of expertise that occur in different planes of motion, e.g., a kick from hand. This is particularly important since visual processing of biological motion is seemingly influenced by the translational plane of motion (Wilson et al., 2010). In addition, the current methodology did not allow the RM effect to be tested with neutral-stimuli, making the RM observations difficult to solely attribute to expertise. That said, Nakamoto et al. (2015) report that sporting expertise does not serve to independently influence neutral stimuli. In addition, the complexity of the study design, the number of levels in the analyses, and the small effect sizes reported, mean results could have occurred by chance alone. However, given the effect of expertise on anticipation reported in the literature, as well as that of visual field asymmetries on the RM effect (c.f., Hubbard and Ruppel, 1999), the findings of the current study do not stand out of context with the body of work reported in the literature. Further research, particularly with paradigms that can appropriately mirror the dynamic nature of sport performance, should aim to clarify this.

## Conclusion

This study is the first to provide support for the TEC as an explanation for action anticipation in expert sport performers, in so much that the acquisition of sporting expertise promotes automatic anticipation of observed actions toward their perceived distal effects. That is, automatic anticipation, as measured by representational momentum, was greater for experts when there was congruency between the observed action and its effect (i.e., when the rugby pass was directed toward a teammate). However, when the link between the observed action and its effect was incongruent (i.e., when the pass was directed away from the teammate), experts did not anticipate and as a result were better able to meet the task demands, i.e., to accurately recall the final position of the passer. The anticipation of novices occurred independent of congruency. These findings suggest experts automatically anticipate if the perceived action and its effect are congruent. Whereas, there was no anticipation in experts when actions were incongruent with their learned effects. These data also point toward a possible expert-specific ability to inhibit automatic anticipation in order to preserve goal-oriented action if actions appear incongruent with their learned effects. These findings likely have implications for an expert’s ability to accurately perceive and respond (or inhibit automatic responses) to deceptive actions. That is, if deceptive actions are based on congruent action-effect contexts, anticipation to the deception may occur automatically in experts. On the other hand, if deceptive actions are made that are incongruent with the context’s typical effect, experts either do not automatically anticipate or may be able to inhibit automatic anticipation. We recommend that future research explicitly investigate inhibition within the above context in order to further examine these proposals.

## Ethics Statement

The study was granted ethical approval by and conducted in accordance with the ethical guidelines of the Ethics Committee of the School of Sport, Health and Exercise Sciences, Bangor University for research involving human participants and in line with the 1964 Helsinki declaration and its later amendments or comparable ethical standards.

## Author Contributions

GL led initial study conceptualisation, design, and set of methods. DA was responsible for data collection, under the supervision of GL and VG and conducted data analysis. All authors subsequently contributed to interpretation of findings. DA provided a first draft of the manuscript which was then edited by GL and VG.

### Conflict of Interest Statement

The authors declare that the research was conducted in the absence of any commercial or financial relationships that could be construed as a potential conflict of interest.
